# Capsaicin-Induced Impairment of Functional Network Dynamics in Mouse Hippocampus via a TrpV1 Receptor-Independent Pathway: Putative Involvement of Na^+^/K^+^-ATPase

**DOI:** 10.1007/s12035-019-01779-3

**Published:** 2019-11-07

**Authors:** Hugo Balleza-Tapia, Pablo Dolz-Gaiton, Yuniesky Andrade-Talavera, André Fisahn

**Affiliations:** Neuronal Oscillations Laboratory, Neurogeriatrics Division, Center for Alzheimer Research, Department of Neurobiology, Care Sciences and Society, Karolinska Institutet, Akademiska stråket 1, 17164 Solna, Sweden

**Keywords:** TrpV1 receptor, Capsaicin, Na^+^/K^+^-ATPase, Hippocampus, Cellular and synaptic physiology, Neuronal network, Gamma oscillations, Functional network dynamics

## Abstract

The vanilloid compound capsaicin (Cp) is best known to bind to and activate the transient receptor potential vanilloid receptor-1 (TrpV1). A growing number of studies use capsaicin as a tool to study the role of TrpV1 in the central nervous system (CNS). Although most of capsaicin’s CNS effects have been reported to be mediated by TrpV1 activation, evidence exists that capsaicin can also trigger functional changes in hippocampal activity independently of TrpV1. Recently, we have reported that capsaicin induces impairment in hippocampal gamma oscillations via a TrpV1-independent pathway. Here, we dissect the underlying mechanisms of capsaicin-induced alterations to functional network dynamics. We found that capsaicin induces a reduction in action potential (AP) firing rate and a subsequent loss of synchronicity in pyramidal cell (PC) spiking activity in hippocampus. Moreover, capsaicin induces alterations in PC spike-timing since increased first-spike latency was observed after capsaicin treatment. First-spike latency can be regulated by the voltage-dependent potassium current D (*I*_D_) or Na^+^/K^+^-ATPase. Selective inhibition of *I*_D_ via low 4-AP concentration and Na^+^/K^+^-ATPase using its blocker ouabain, we found that capsaicin effects on AP spike timing were completely inhibited by ouabain but not with 4-AP. In conclusion, our study shows that capsaicin in a TrpV1-independent manner and possibly involving Na^+^/K^+^-ATPase activity can impair cognition-relevant functional network dynamics such as gamma oscillations and provides important data regarding the use of capsaicin as a tool to study TrpV1 function in the CNS.

**Electronic supplementary material:**

The online version of this article (10.1007/s12035-019-01779-3) contains supplementary material, which is available to authorized users.

## Introduction

Rhythmic neuronal network activity such as gamma oscillations (30-80 Hz) are crucial for information processing in the brain [1] and emerge from the synchronization of the action potential firing patterns of different neuron types as a result of the coordinated interaction between excitation and inhibition in a neuronal network [2, 3]. Gamma oscillations have been suggested to underlie cognitive processes such as attention, sensory perception and memory [4–6], and alterations of this brain rhythm have been found to be associated with the decline of cognitive function in neurodegenerative pathologies such as Alzheimer’s disease [7, 8] and Parkinson’s disease [9].

Capsaicin (Cp), the active component of chili peppers, is a member of the vanilloid compound family [10] and is best known to bind and activate the transient receptor potential vanilloid receptor-1(TrpV1) [11]. Capsaicin has been widely used to study nociception and sensory transmission in the peripheral nervous system, which has high TrpV1 expression in primary sensory afferents [12]. Despite most of Cp’s effects on sensory neurons being dependent on activation of TrpV1, some studies have reported that Cp can also drive effects through mechanisms independent of TrpV1 activation. Such mechanisms include the regulation of voltage-gated sodium (VGSC) and potassium (VGPC) channel activity [13–15].

Due to the ability to bind to and activate TrpV1, Cp has been used as a tool to study the role of TrpV1 in the central nervous system (CNS), including the modulation of synaptic transmission and plasticity [12, 16, 17]. Although most Cp effects on hippocampal physiology have been demonstrated to be dependent on TrpV1 activation [18–22], some studies have shown that similar Cp concentrations used to study TrpV1 in the hippocampus can also impair synaptic and neuronal network activity independently of TrpV1 activation. For instance, Benninger and coworkers (2008) [23] reported that Cp induced alterations on evoked and spontaneous excitatory postsynaptic currents (EPSC) in granule cells of the dentate gyrus in mice lacking TrpV1 (TrpV1 KO). Similarly, by using the TrpV1-antagonist capsazepine (Cz), we recently have shown that Cp impairs hippocampal neuronal network function by reducing gamma oscillation power in area CA3 through a mechanism independent of TrpV1 activation [24]. In this study, we aim to uncover the underlying mechanisms of the TrpV1-independent Cp-induced alterations to this cognition-relevant functional network dynamic.

## Material and Methods

### Drugs and Chemicals

All chemical compounds used in extracellular and intracellular solutions as well as 4-aminopyridine (4-AP), ouabain octahydrate (ouabain), and nifedipine were obtained from Sigma-Aldrich Sweden AB (Stockholm, Sweden). Kainic acid (KA), (*E*)-capsaicin (Cp), capsazepine (Cz), d-(-)-2-amino-5-phosphonopentanoic acid (AP5), 6,7-dinitroquinoxaline-2,3-dione disodium salt (DNQX), picrotoxin, *N*-(2,6-Dimethylphenylcarbamoylmethyl)-triethylammonium bromide (QX-314), and tetrodotoxin citrate (TTX) were obtained from Tocris Bioscience (Bristol, UK). Ouabain was dissolved in standard ACSF (composition see below) and incubated 1 h at 37 °C before use. 4-AP, nifedipine, Cp, Cz, and picrotoxin were dissolved in DMSO 100%. AP5, DNQX, TTX, and KA were dissolved in milliQ water.

### Animals

Experiments were performed in accordance with the ethical permit granted by Norra Stockholms Djurförsöksetiska Nämnd to AF (N45/13). Animals used in this study included p18-35 C57BL/6 (WT) and TrpV1 knockout (TrpV1 KO) male mice (Charles River Laboratories and Jackson Laboratory, respectively). Animals were deeply anesthetized using isoflurane before being sacrificed by decapitation.

### Hippocampal Slice Preparation

To prepare hippocampal slices, the brain was dissected out and placed in ice-cold artificial cerebrospinal fluid (ACSF) modified for dissection containing (in mM) 80 NaCl, 24 NaHCO_3_, 25 glucose, 1.25 NaH_2_PO_4_, 1 ascorbic acid, 3 Na pyruvate, 2.5 KCl, 4 MgCl_2_, 0.5 CaCl_2_, 75 sucrose, and bubbled with carbogen (95% O_2_ and 5% CO_2_). Horizontal sections (350 μm thick) of the ventral hippocampi of both hemispheres were cut with a Leica VT1200S vibratome (Leica Microsystems). After cutting, slices were transferred to a humidified interface holding chamber containing standard ACSF (in mM): 124 NaCl, 30 NaHCO_3_, 10 glucose, 1.25 NaH_2_PO_4_, 3.5 KCl, 1.5 MgCl_2_, 1.5 CaCl_2_, continuously supplied with humidified carbogen. The chamber was held at 37 °C in a water bath during slicing and subsequently allowed to cool down to room temperature for at least 60 min before any experimental manipulation.

### Electrophysiology

Local field potential recordings (LFPs) were performed in a submerged recording chamber using glass microelectrodes (4–6 MΩ) filled with standard ACSF and placed in CA3 *stratum pyramidale*. Patch clamp (whole-cell) recordings were performed with borosilicate glass microelectrodes (4–6 MΩ) from visually identified CA3 pyramidal cells (PCs) using IR-DIC microscopy (Zeiss Axioskop, Germany and BX50WI Olympus, Japan). For action potential (AP) firing and sEPSC recordings (Vh = −70 mV), a potassium-based intracellular solution was used (in mM): 122.5 K-gluconate, 8 KCl, 4 Na_2_ATP, 0.3 NaGTP, 10 HEPES, 0.2 EGTA, 2 MgCl_2_, set to pH 7.2–7.3 with KOH. For sIPSC recordings (Vh = −70 mV), a high-chloride cesium-based solution was used (in mM): 135 CsCl, 10 HEPES, 0.2 EGTA, 4 MgCl_2_, 2 Na_2_ATP, 0.2 NaGTP, 5 QX-314, set to pH 7.2–7.4 with CsOH; 50 μM picrotoxin was applied at the end of these experiments. For potassium current I-V curves, the K-gluconate-based solution was used but EGTA content increased to 9 mM. In addition, 1 μM TTX and 1 μM nifedipine were bath-applied to block voltage-gated sodium and calcium channels, respectively. Data were recorded with MultiClamp 700B and Axopatch 200B amplifiers (Molecular Devices), sampled at 10 kHz, conditioned using a HumBug 50 Hz noise eliminator (LFP signals only; Quest Scientific), low-pass filtered at 2 kHz, digitized (Digidata 1440A, Molecular Devices, CA, USA) and stored on a hard disc using pCLAMP 10.4 software (Molecular Devices). Gamma oscillations were elicited by applying 100 nM KA to the extracellular bath and recorded at 34 °C [25]. Oscillations were allowed to stabilize for at least 20 min before recording.

### Data Analysis

Fast Fourier transformations for power spectra were calculated from 1-min-long LFP data traces (segments of 8192 points) using Clampfit 10.7 software (Molecular Devices). Gamma oscillation power was calculated as the integrated power spectrum between 20 and 80 Hz. Auto-correlograms were calculated in Clampfit using a 100-ms lag from the same LFP trace used for gamma power calculation. All LFP traces were pre-processed by applying a bandpass filter set to 15–40 Hz (RC-single pole) using Clampfit software.

Coefficient of rhythmicity (Cr) was calculated as a measure of the quality of gamma oscillations [24, 26, 27] and was defined as Cr = (α-β)/(α + β) with α corresponding to the value of the height of the second peak and β to the value of the depth of the first trough in the normalized auto-correlogram. Cr ranges from 0 to 1 with higher numbers denoting better rhythmicity in the analyzed activity. Only recordings having a Cr ≥ 0.01 were considered rhythmic. In all cases, the rhythm frequency predicted by the auto-correlogram was confirmed with the power spectrum of each recording segment analyzed.

sEPSC and sIPSC were detected offline using MiniAnalysis software (Synaptosoft, Decatur, GA, USA). Charge transfer, event amplitude and inter-event-interval (IEI) were analyzed using Excel software (Microsoft Office) and GraphPad Prism (GraphPad Software, USA) with the results representing averaged values taken over 1-min periods.

Spike phase-coupling analysis was performed on concomitant LFP and single-cell recordings using Matlab custom-written routines (code accessibility) in order to relate the PC spiking activity to ongoing gamma oscillations as previously reported [24, 28]. To do this, 5-min-long segments from control and experimental condition recordings were used to perform the analysis. LFPs were pre-processed using a bandpass filter set to 15–50 Hz (RC-single pole) in Clampfit. APs were detected by setting an amplitude threshold, and the instantaneous phase of gamma oscillation was calculated using a Hilbert transform in order to determine the phase angle at which each action potential occurred during ongoing oscillations.

Phase angles and gamma oscillation phases were represented in polar plots and expressed in radians with the peak of the oscillation cycle corresponding to 0π and the trough corresponding to π in the polar plots. In order to determine the synchronization level of AP firing, the phase angles of the APs were calculated by vector averaging. Considering that each AP has a phase angle and can therefore be described as a vector with length 1, the resultant average vector length represents the synchronization level of AP firing and ranges from 0 to 1, with 0 representing PC firing uniformly distributed throughout the oscillation cycle and 1 representing PC firing at the same phase angle only. All recordings were tested for circular uniformity using Rayleigh’s test. Only recordings with non-uniform circular distribution (*p* < 0.05) were considered for analysis.

Potassium current I–V curves were performed by applying an 11-step protocol with 10 mV increments in voltage-clamp whole-cell configuration on PCs (see insert in Fig. 3e). Measurements were done in the steady state of the current to calculate the I–V relationship.

First-spike latency measurements were done by applying a rheobase protocol on PCs in current-clamp whole-cell configuration (increasing 10 pA current steps of 300 ms to elicit the firing of a single action potential). First-spike latency was then calculated as the time between the beginning of the test current pulse and the maximum amplitude of the AP.

### Experimental Design and Statistical Analysis

A complete description of the experimental design of each individual experiment can be found in the “Results” section. Each “*n*” within an experiment represents an individual hippocampal slice and/or cell, and a minimum of three animals were used per experiment.

All statistical analyses were performed using GraphPad Prism. Time-course data are presented normalized for comparison purposes between the different experimental conditions. For this, data were binned and analyzed over 1-min periods and then normalized to the average of 5 min of control recording before treatment application. Tests for statistical significance were performed on absolute values in all experiments using Wilcoxon signed rank test (one tailed) for paired data and Mann–Whitney *U* test (one tailed) for unpaired data. Significance was set at *p* = 0.05 for all statistical analyses. Data are presented as mean ± SEM. * indicates *p* < 0.05 and **p < 0.01.

## Results

### Capsaicin (Cp) Impairs Gamma Oscillations Through a TrpV1-Independent Mechanism

**Fig. 1:**
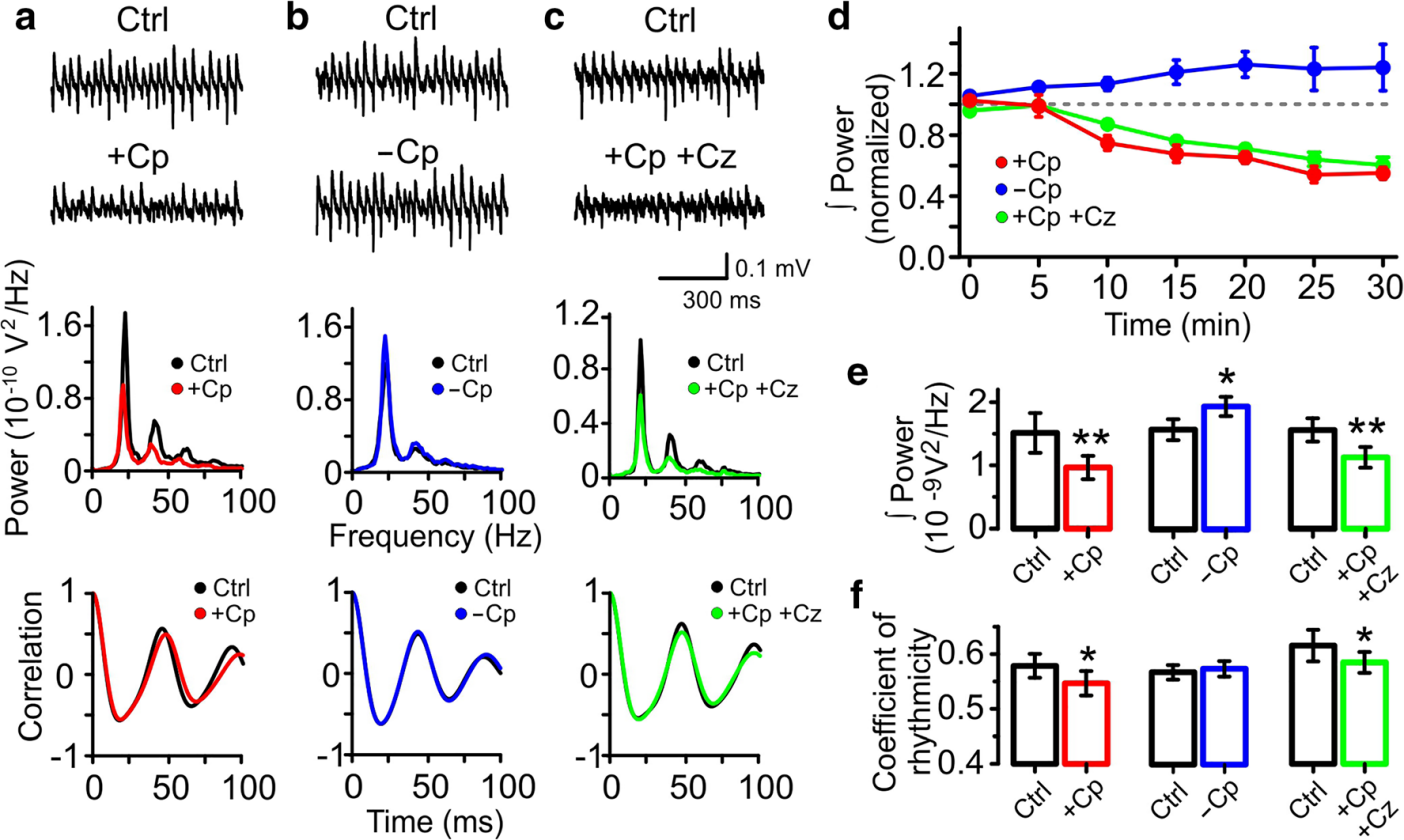
Capsaicin (Cp) impairs gamma oscillation through a mechanism independent of TrpV1 activation. **a**–**c** Representative sample traces (top) power spectra (middle) and auto-correlograms (bottom) of KA-induced gamma oscillations in hippocampal slices treated with 20 μM capsaicin (+Cp, red), control slices without Cp treatment (−Cp, blue), and slices treated with 20 μM Cp together with 20 μM capsazepine (+Cp +Cz, green). **d** Time-course of the integrated power of gamma oscillations from the experimental conditions described in **a** showing the time-dependent decrease in gamma power in slices treated with Cp (red) and in the slices treated with Cp plus capsazepine (green). **e**, **f** Summary bar graph of the integrated gamma power and coefficient of rhythmicity (Cr), respectively, from the experimental conditions described in **a**–**c**. Note that Cp-induced decrease of gamma oscillations and Cr could not be reversed by capsazepine, showing that TrpV1 receptor activation is not involved. Integrated power was measured on 1-min segments every 5 min after treatment application. Power quantification was performed after 20 min of treatment application and compared to the average of 5 min of control activity. Cr quantification was performed between 20 min time point and 1 min of control activity before treatment application. Wilcoxon signed rank test (one-tailed) was used for statistical significance on absolute values. Data is presented as mean ± SEM. *p < 0.05; **p < 0.01

Recently, we have reported that hippocampal slices pre-incubated with 10 μM Cp displayed reduced gamma oscillation power due to activation of a TrpV1-independent mechanism [24]. To further explore the TrpV1-independent effect of Cp on hippocampal network function, we tested first the effect of 20 μM Cp on ongoing gamma oscillations. We tested this higher Cp concentration in order to isolate and focus on the Cp TrpV1-independent effects [14, 15, 23, 29, 30]. Gamma oscillations were recorded at 34 °C and elicited by bath application of 100 nM KA. After 20 min KA application, 20 μM Cp was bath perfused and the effect on gamma power was monitored over time (Fig. 1, *n* = 7). We found that Cp treatment reduced gamma oscillation power in a time-dependent manner (Fig. 1d; Table 1). Cp induced a significant reduction in gamma power after 10 min, reaching the maximum effect at 25 min after Cp application (Fig. 1d; Table 1).

**Table 1:** Summary of the time course of gamma oscillation power shown in Fig. 1d

Time (min)	Integrated gamma power (10^−9^ V^2^)				
	+Cp (*n* = 7)	−Cp (*n* = 6)	+Cp +Cz (*n* = 7)	+Cz (*n* = 6)	+DMSO (*n* = 5)
Average (−5 to −1)	1.51 ± 0.31	1.58 ± 0.17	1.56 ± 0.18	0.95 ± 0.10	2.22 ± 0.18
0	1.54 ± 0.31 (*p* = 0.2344)	*1.66 ± 0.16 (*p* = 0.0156)	1.51 ± 0.19 (*p* = 0.0547)	0.97 ± 0.12 (*p* = 0.2813)	2.29 ± 0.16 (*p* = 0.0313)
5	1.41 ± 0.26 (*p* = 0.1484)	*1.73 ± 0.14 (*p* = 0.0156)	1.55 ± 0.18 (*p* = 0.4688)	0.93 ± 0.16 (*p* = 0.4219)	2.33 ± 0.17 (*p* = 0.0625)
10	**1.09 ± 0.22 (*p* = 0.0078)	*1.77 ± 0.17 (*p* = 0.0156)	**1.37 ± 0.18 (*p* = 0.0078)	1.04 ± 0.19 (*p* = 0.3438)	2.35 ± 0.23 (*p* = 0.1563)
15	**1.01 ± 0.20 (*p* = 0.0078)	*1.87 ± 0.15 (*p* = 0.0313)	**1.20 ± 0.17 (*p* = 0.0078)	1.03 ± 0.22 (*p* = 0.5)	2.35 ± 0.21 (*p* = 0.3125)
20	**0.97 ± 0.18 (*p* = 0.0078)	*1.94 ± 0.15 (*p* = 0.0156)	**1.13 ± 0.16 (*p* = 0.0078)	1.10 ± 0.21 (*p* = 0.3438)	2.49 ± 0.25 (*p* = 0.0625)
25	**0.86 ± 0.19 (*p* = 0.0078)	1.88 ± 0.18 (*p* = 0.1094)	**1.02 ± 0.16 (*p* = 0.0078)	1.07 ± 0.26 (*p* = 0.4219)	2.46 ± 0.27 (*p* = 0.2188)
30	**0.86 ± 0.18 (*p* = 0.0078)	1.91 ± 0.24 (*p* = 0.0781)	**0.96 ± 0.17 (*p* = 0.0078)	1.14 ± 0.33 (*p* = 0.5)	2.57 ± 0.29 (*p* = 0.0938)

For statistical analysis, the value of each time point was compared to the average of its own 5 min of control activity. Wilcoxon signed rank test (one-tailed) was used for statistical significance on absolute values. Data is presented as mean ± SEM

*p < 0.05; **p < 0.01

For comparison purposes across all experiments, we decided to use the time point 20 min after Cp application since our previous study found Cp effects between 15 and 20 min. Capsaicin (20 μM) induced a 34.74 ± 3.72% reduction in gamma oscillation power (control = 1.51 ± 0.31 × 10^−9^ V^2^; +Cp = 0.97 ± 0.18 × 10^−9^ V^2^; *p* = 0.0078; *n* = 7; Fig. 1). The power reduction correlated with a degradation of the quality of gamma oscillations seen as a reduction in the coefficient of rhythmicity (control Cr = 0.58 ± 0.02; +Cp Cr = 0.55 ± 0.02; *p* = 0.0156; Fig. 1f). In contrast, at the same 20-min time point in slices without Cp treatment (−Cp; Fig. 1b, e, f), gamma oscillations showed no reduction in power (control = 1.58 ± 0.17 × 10^−9^ V^2^; −Cp = 1.94 ± 0.15 × 10^−9^ V^2^; *p* = 0.0156; *n* = 6) and rhythmicity (control Cr = 0.56 ± 0.01; −Cp Cr = 0.57 ± 0.01; *p* = 0.2188; Fig. 1f). Moreover, gamma oscillations displayed a small increase in power over time in the slices not treated with Cp (Fig. 1d, e; Table 1).

To test whether the TrpV1 receptor was involved in Cp effects on gamma oscillations, we applied 20 μM Cp together with 20 μM of the TrpV1 receptor antagonist capsazepine. The results showed that the combined treatment also induced a time-dependent reduction of gamma oscillation power (Fig. 1d, *n* = 7; Table 1). Reduction in power (control = 1.56 ± 0.18 × 10^−9^ V^2^; +Cp +Cz = 1.13 ± 0.16 × 10^−9^ V^2^; *p* = 0.0078; *n* = 7; Fig. 1c, e) and rhythmicity of gamma oscillations (control = 0.62 ± 0.03; +Cp +Cz = 0.58 ± 0.02; *p* = 0.0391; Fig. 1c, f) following combined treatment were similar to the values observed in the slices treated with Cp only (+Cp +Cz = 29.0 ± 3.5% reduction). In an additional control experiment, we treated slices with 20 μM capsazepine only and, as expected, gamma oscillations displayed a similar time course to slices without Cp treatment (+Cz, Fig. S1C, *n* = 6, Table 1). No effect was found on gamma oscillation power (control = 0.95 ± 0.1 × 10^−9^ V^2^; +Cz = 1.10 ± 0.21 × 10^−9^ V^2^; *p* = 0.3438; *n* = 6; Fig. S1A, D) or rhythmicity (control = 0.63 ± 0.01; +Cz = 0.64 ± 0.01; *p* = 0.5; Fig. S1A, D) after capsazepine-only treatment. Similar results were obtained in hippocampal slices treated with 0.05% DMSO (Cp andCz vehicle; Fig. S1C; Table 1): no changes were observed in gamma power (control = 2.22 ± 0.18 × 10^−9^ V^2^; DMSO = 2.49 ± 0.25 × 10^−9^ V^2^; *p* = 0.0625; *n* = 5; Fig. S1B, D) or rhythmicity (control = 0.64 ± 0.04; DMSO = 0.66 ± 0.04; *n* = 5; p = 0.0625; Fig. S1B, D). Taken together, these data indicate that 20 μM Cp impairs gamma oscillations by altering the rhythmicity of hippocampal activity through a mechanism independent of TrpV1 receptor activation.

### Capsaicin (Cp)-Induced Impairment of Gamma Oscillations Is Caused by a Desynchronization of Pyramidal Cell Activity

**Fig. 2:**
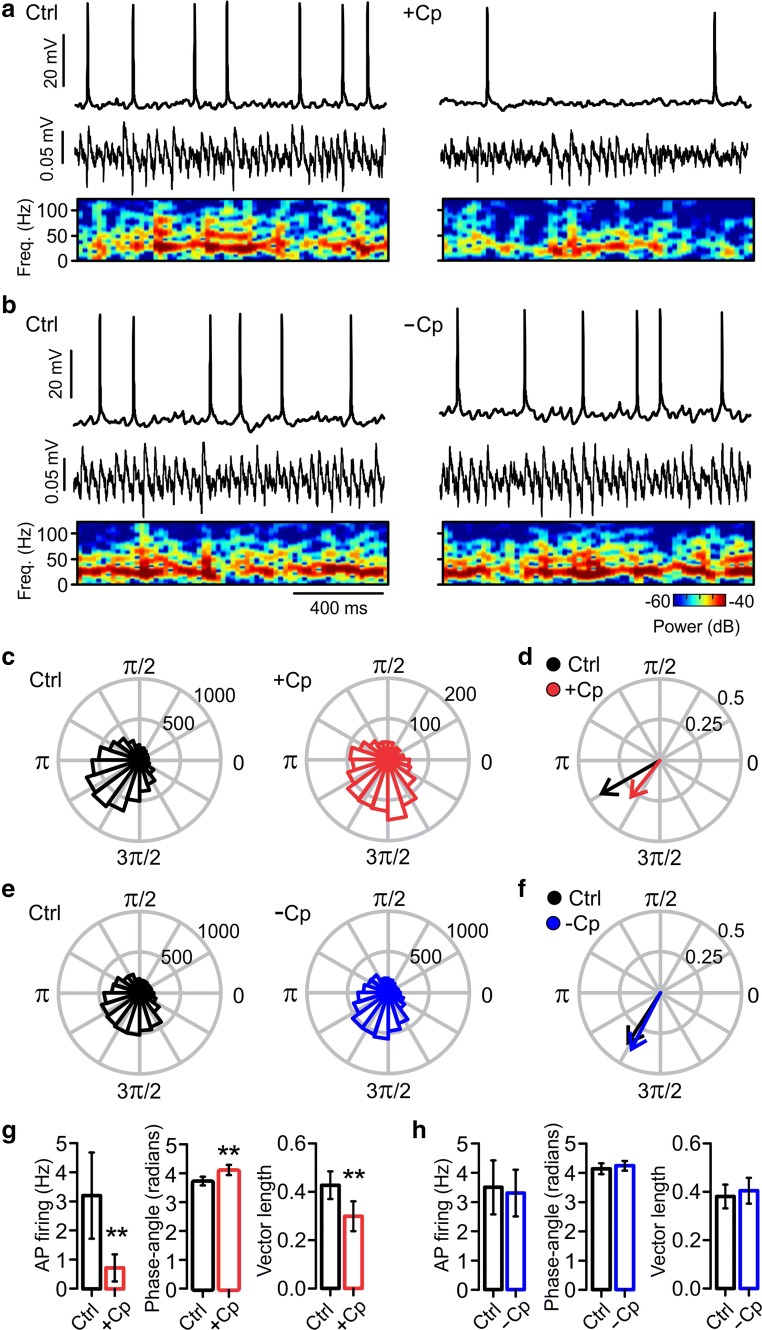
Capsaicin (Cp)-induced reduction of gamma oscillations is caused by a desynchronization of PC activity. **a** Representative sample traces of concomitant LFP and patch-clamp recordings (top and middle) of CA3 PCs showing the Cp-induced reduction (+Cp) in gamma oscillation power (spectrogram, bottom) and AP firing rate. **b** Representative sample traces of concomitant LFP and patch-clamp recordings (top and middle) as well as gamma power (spectrogram, bottom) from control slices without Cp treatment (−Cp). **c**, **e** Polar plots showing the distribution of the total AP phase angles from slices before and after 20 μM Cp application (+Cp) and from control slices without Cp (−Cp). Phase angles and gamma oscillation phases are presented in radians; the peak of the oscillation cycle corresponds to 0π and the trough corresponds to π. **d**, **f** Resultant average vector showing the preferred phase angle (arrow direction) and the synchronization level of AP firing (vector length) from slices treated with Cp (+Cp) and control slices without Cp treatment (−Cp). **g**, **h** Summary bar graphs of the AP firing rate, phase angles, and vector length in slices before (black) and after 20 μM Cp application (+Cp, red) and from slices without Cp treatment (−Cp, blue). Note that in parallel with the reduction in gamma oscillation power (spectrogram), the Cp induced a reduction of AP firing rate that correlates with a loss of synchronicity of PC spiking activity. Quantifications were performed on 5-min segments of concomitant recordings from control activity and between 16 and 20 min after Cp application. Same time points from slices without Cp treatment. Wilcoxon signed rank test (one-tailed) was used for statistical significance on absolute values. Data is presented as mean ± SEM. **p < 0.01

Because Cp impaired the rhythmicity of gamma oscillation, our next experiments focused on testing Cp’s effect on AP-phase coupling of PC AP firing during gamma oscillations (see “Materials and Methods”). To do this, we recorded AP firing of PCs using whole-cell current-clamp configuration concomitantly with LFP recordings of gamma oscillations. Control activity over 5 min was compared to activity between 15 and 20 min after Cp application to improve the accuracy of AP-phase coupling analysis. The results showed that Cp reduced AP firing rate (control = 3.21 ± 1.48 Hz; +Cp = 0.72 ± 0.47 Hz; *p* = 0.0078; *n* = 7; Fig. 2a, g) in parallel to the reduction of gamma oscillation power (Fig. 2a). In contrast, there were no changes in AP firing rate (control = 3.50 ± 0.92 Hz; −Cp = 3.30 ± 0.80 Hz; *p* = 0.3438; *n* = 6; Fig. 2b, h) as well as gamma oscillation power in slices without Cp application (−Cp; Fig. 2b). Together with the reduction in AP firing and gamma oscillation power, Cp also induced a significant change in the preferred AP-phase angle (control = 3.66 ± 0.15 rad; +Cp = 4.04 ± 0.17 rad; *p* = 0.0078; *n* = 7; Fig. 2c, d, g), observed as a small shift towards higher angle values in the AP-phase angle distribution window and resultant vector (Fig. 2c, d).

Importantly, our results showed that Cp induced a desynchronization of PC activity that can be observed as a reduction in the vector length of AP-phase coupling (control = 0.43 ± 0.06; +Cp = 0.30 ± 0.06; *p* = 0.0078; *n* = 7; Fig. 2d, g) and a slight increase in the width of the AP-phase distribution window (Fig. 2c). As expected, in slices without Cp treatment (−Cp), no changes were found in the preferred AP-phase angle (control = 4.14 ± 0.18 rad; −Cp = 4.24 ± 0.17 rad; *p* = 0.3438, *n* = 6, Fig. 2e, h) and vector length (control = 0.38 ± 0.05; −Cp = 0.40 ± 0.05; *p* = 0.2813; *n* = 6; Fig. 2f, h). Our results suggest that Cp impairs gamma oscillations by inducing a reduction in AP firing rate and a desynchronization of PC activity in the hippocampal network.

### Cp Induces a Reduction in AP-Firing Rate in a Non-Activated Neuronal Network

**Fig. 3:**
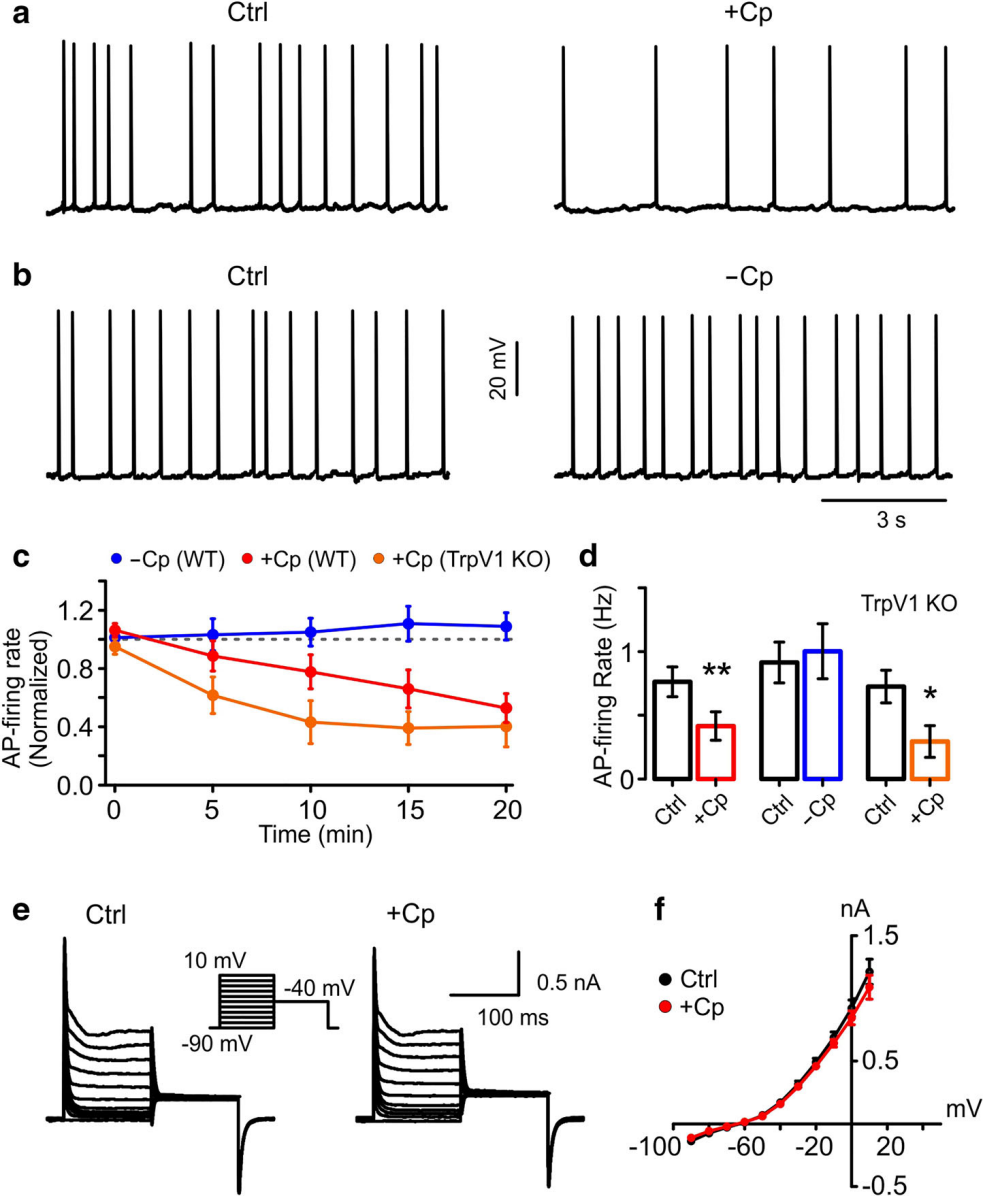
Capsaicin (Cp)-induced reduction in AP-firing rate is not caused by modulation of potassium currents. **a** Representative recordings of the AP-firing rate of PCs from slices not activated with KA showing that 20 μM Cp (+Cp, 20 min) induced a reduction in the firing rate similar to PCs in slices activated with KA. **b** PC AP firing rate from control slices without Cp treatment (20 min). **c** Time-course of AP firing rate in slices treated with 20 μM Cp (+Cp) from wild-type mice (WT, red) and from TrpV1 knockout mice (TrpV1 KO, orange). Also shown is the time-course of AP firing rate from control WT slices without Cp treatment (−Cp, blue). **d** Summary bar graph of the experimental conditions described in **c** after 20 min of 20 μM Cp treatment (+Cp, WT red, TrpV1 KO orange) and the same time point for control slices not treated with Cp (−Cp, blue). **e** Representative recordings of potassium currents elicited in PCs before (Ctrl, black) and after 20 μM Cp treatment (+Cp, 20 min, red). The insert shows the pulse protocol used. **f** I-V curves of the potassium currents recorded in **f**. Note that the Cp-induced reduction of AP firing rate is not related to a modulation of potassium currents. AP-firing rate quantification was performed on 1-min segments after 20 min of treatment application and compared to the average of 5 min of control activity. Wilcoxon signed rank test (one-tailed) was used for statistical significance on absolute values. Data is presented as mean ± SEM. *p < 0.05; **p < 0.01

In order to isolate and study the mechanism underlying the Cp-induced reduction in AP firing rate, we performed experiments on quiescent slices (network not activated by KA) to remove the influence of KA on the hippocampal network. For this, we recorded the AP firing rate of PCs in whole-cell current-clamp configuration at room temperature. In order to simulate the PC spiking activity during gamma oscillations, we injected current (*I*_inj_) into PCs to obtain a stable firing frequency. Using this approach, we found that 20 μM Cp induced a reduction in the AP firing rate of PCs (control = 0.76 ± 0.12 Hz; +Cp = 0.42 ± 0.11 Hz; *p* = 0.0029; *n* = 10; *I*_inj_ = 48.6 ± 5.6 pA; Fig. 3a, d). This reduction was time-dependent (Fig. 3c; Table 2), similarly to the reduction in gamma oscillation power shown previously (Fig. 1d, Table 1). In contrast, slices without Cp treatment displayed no reduction in AP firing rate (control = 0.92 ± 0.16 Hz; −Cp = 1.0 ± 0.22 Hz; *p* = 0.3125; *n* = 5; *I*_inj_ = 52.0 ± 5.1pA; Fig. 3b–d; Table 2).

**Table 2:** Summary of the time course of AP firing rate shown in Fig. 3c

Time (min)	AP-firing rate (Hz)			
	+Cp (*n* = 10)	−Cp (*n* = 5)	+Cp (TrpV1 KO) (*n* = 7)	+Cp (34 °C) (*n* = 7)
Average (−5 to −1)	0.76 ± 0.12	0.92 ± 0.16	0.73 ± 0.13	0.93 ± 0.14
0	0.79 ± 0.12 (*p* = 0.1539)	0.92 ± 0.16 (*p* = 0.3125)	0.68 ± 0.12 (*p* = 0.1484)	0.96 ± 0.13 (*p* = 0.2891)
5	0.71 ± 0.17 (*p* = 0.3125)	0.91 ± 0.14 (*p* = 0.4063)	*0.46 ± 0.13 (*p* = 0.0156)	0.68 ± 0.11 (*p* = 0.0547)
10	0.60 ± 0.13 (*p* = 0.0527)	0.95 ± 0.17 (*p* = 0.3125)	*0.33 ± 0.13 (*p* = 0.0156)	*0.56 ± 0.09 (*p* = 0.0234)
15	**0.51 ± 0.12 (*p* = 0.0098)	1.01 ± 0.21 (*p* = 0.1393)	**0.30 ± 0.12 (*p* = 0.0078)	*0.51 ± 0.08 (*p* = 0.0156)
20	**0.42 ± 0.11 (*p* = 0.0029)	1.00 ± 0.22 (*p* = 0.3125)	*0.29 ± 0.13 (*p* = 0.0156)	*0.44 ± 0.09 (*p* = 0.0156)

For statistical analysis, the value of each time point was compared to the average of its own 5 min of control activity. Wilcoxon signed rank test (one-tailed) was used for statistical significance on absolute values. Data is presented as mean ± SEM

*p < 0.05; **p < 0.01

To corroborate that under these experimental conditions Cp’s effect was TrpV1-independent, additional recordings were performed in hippocampal slices from TrpV1 KO mice. The results showed that Cp, similar to its action in WT slices, still induced a time-dependent reduction of the PC AP firing rate in TrpV1 KO slices (control = 0.73 ± 0.13 Hz; +Cp = 0.29 ± 0.13 Hz; *p* = 0.0156; *n* = 7; *I*_inj_ = 50.0 ± 8.7 pA; Fig. 3c, d; Table 2). In addition, to control for differences in the recording temperature compared to the experiments using KA, some recordings were made at 34 °C and found that Cp reduced AP firing (control = 0.93 ± 0.14 Hz; +Cp = 0.44 ± 0.09 Hz; *p* = 0.0156; *n* = 7; *I*_inj_ = 62.7 ± 6.1 pA; Fig. S2A–C; Table 2) similar to the experiments performed at room temperature. Taken together, our data indicate that the Cp effect on hippocampus is not dependent on the activation state of the neural network.

Alterations to AP firing could be caused by modulation of potassium currents. Consequently, we tested whether Cp could alter potassium current conductance. To do this, we performed whole-cell recordings in voltage-clamp configuration to record potassium currents in hippocampal CA3 PCs before and after Cp treatment (Fig. 3e). We found that Cp did not induce any observable alterations in the potassium current I–V relationship compared to control conditions (Fig. 3e, f; Table 3), indicating that the Cp-induced reduction of AP firing rate is not caused by a modulation of potassium current conductance in PCs.

**Table 3:** Summary of the potassium current IV curves shown in Fig. 3f

K-currents IV curve			
Vh (mV)	Control (nA)	+Cp (nA)	*p* value
− 90	− 0.14 ± 0.006	− 0.11 ± 0.016	*p* = 0.0625
− 80	− 0.07 ± 0.009	− 0.06 ± 0.011	*p* = 0.0938
− 70	− 0.03 ± 0.011	− 0.02 ± 0.008	*p* = 0.5
− 60	0.01 ± 0.011	0.02 ± 0.007	*p* = 0.3125
− 50	0.07 ± 0.011	0.06 ± 0.007	*p* = 0.1563
− 40	0.17 ± 0.015	0.16 ± 0.008	*p* = 0.3125
− 30	0.32 ± 0.025	0.30 ± 0.010	*p* = 0.3125
− 20	0.49 ± 0.034	0.46 ± 0.017	*p* = 0.2188
− 10	0.69 ± 0.044	0.64 ± 0.031	*p* = 0.2188
0	0.92 ± 0.066	0.85 ± 0.056	*p* = 0.2188
10	1.21 ± 0.101	1.09 ± 0.096	*p* = 0.2188

For statistical analysis, values were compared between each Vh value (Control vs. +Cp). Wilcoxon signed rank test (one-tailed) was used for statistical significance on absolute values. Data is presented as mean ± SEM

### Cp Decreases Inhibitory but Not Excitatory Synaptic Input to PCs

**Fig. 4:**
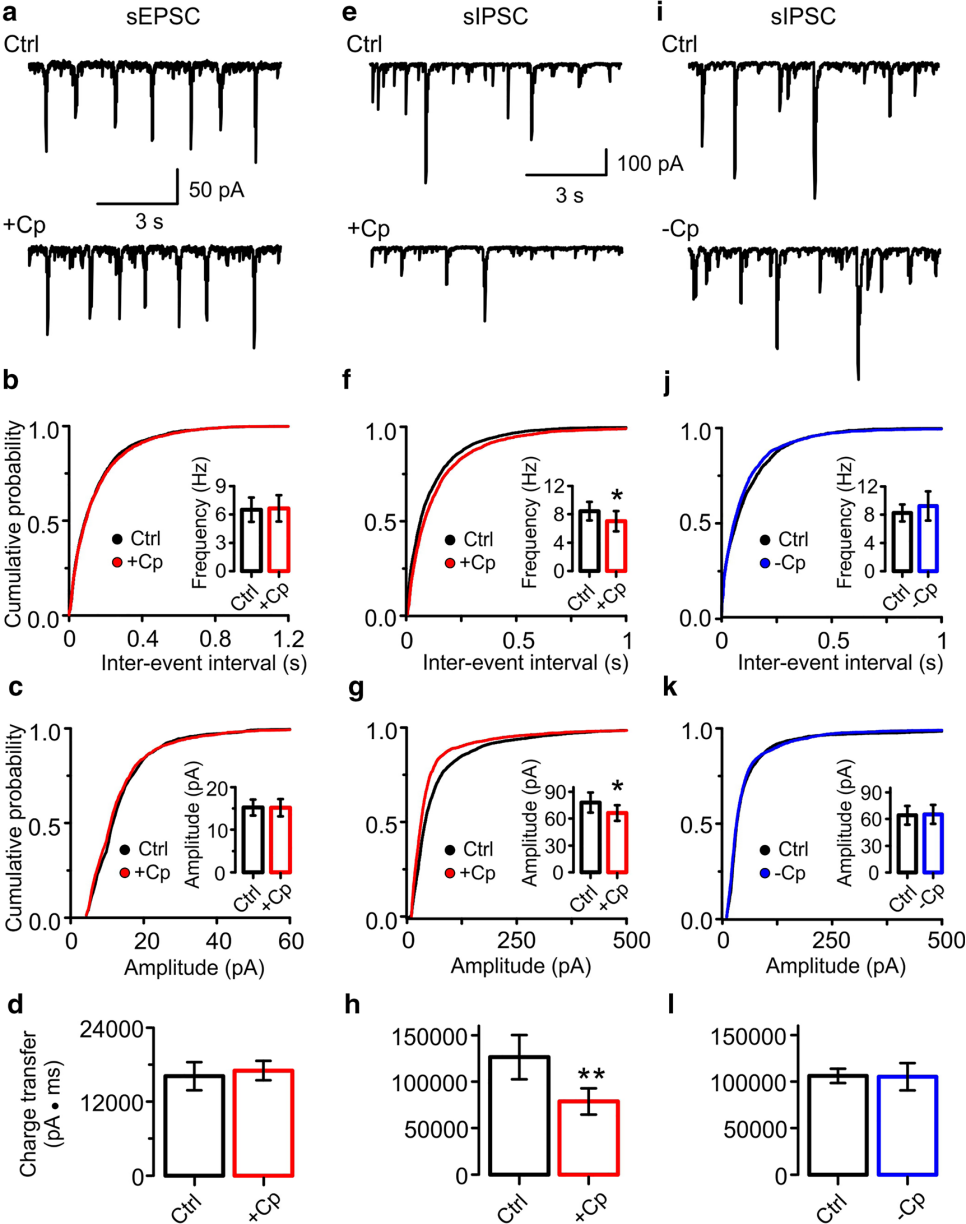
Capsaicin (Cp) decreases inhibitory but not excitatory synaptic input to PCs. **a** Representative traces of sEPSC recorded from PCs (Vh = −70 mV) before and after 20 μM Cp treatment (+Cp, 20 min). **b**, **c** Cumulative probability graph of sEPSC inter-event interval and amplitude (respectively), before (Ctrl, black) and after 20 μM Cp treatment (+Cp, 20 min, red). The inserts show the summary bar graphs of sEPSC frequency and amplitude quantifications. **d** Summary bar graphs of sEPSC charge transfer before (Ctrl, black) and after 20 μM Cp treatment (+Cp, red). **e** Representative traces of sIPSC recorded from PCs (Vh = −70 mV) before and after 20 μM Cp treatment (+Cp, 20 min). **f**, **g** Cumulative probability graph of sIPSC inter-event interval and amplitude (respectively), before (Ctrl, black) and after 20 μM Cp treatment (+Cp, 20 min, red). The inserts show the summary bar graphs of sIPSC frequency and amplitude quantifications. **h** Summary bar graphs of sIPSC charge transfer before (Ctrl, black) and after 20 μM Cp treatment (+Cp, red). **i** Representative traces of sIPSC from PCs in control slices without Cp treatment (−Cp) at the same time points as slices in **e** (Ctrl, black; 20 min, blue). **j**, **k** Cumulative probability graph of sIPSC inter-event interval and amplitude (respectively) of the experimental conditions describe in **i** (Ctrl, black; −Cp, 20 min, blue). The inserts show the summary bar graphs of sIPSC frequency and amplitude quantifications. **l** Summary bar graphs of sIPSC charge transfer in control slices without Cp treatment (−Cp). Quantifications were performed on 1-min segments after 20 min of treatment application and compared to the average of 5 min of control activity. Wilcoxon signed rank test (one-tailed) was used for statistical significance on absolute values. Data is presented as mean ± SEM. *p < 0.05; **p < 0.01

Once we demonstrated that the Cp effect was not dependent on the activation level of the network, we continued our investigation with testing whether Cp could impair the synaptic input to PCs. First, we tested the excitatory input by recording the spontaneous excitatory postsynaptic currents (sEPSC) before and after Cp application. As shown in Fig. 4, Cp (20 μM) did not impair the excitatory input since no alterations were found in sEPSC frequency (control = 6.49 ± 1.29 Hz; +Cp = 6.63 ± 1.40 Hz; *p* = 0.4063; *n* = 5; Fig. 4a, b), amplitude (control = 15.23 ± 1.88pA; +Cp = 15.18 ± 2.04pA; *p* = 0.4063; *n* = 5; Fig. 4a, c) or charge transfer (control = 16,150 ± 2285 pA·ms; +Cp = 17,040 ± 1578 pA·ms; *p* = 0.1563; *n* = 5; Fig. 4a, d).

In contrast, we found a significant reduction in the frequency (control = 8.44 ± 1.32 Hz; +Cp = 7.02 ± 1.43 Hz; *p* = 0.0234; *n* = 7; Fig. 4e, f), amplitude (control = 77.95 ± 11.27 pA; +Cp = 66.21 ± 8.79 pA; *p* = 0.0234; *n* = 7; Fig. 4e, g), and charge transfer (control = 126,400 ± 23890 pA·ms; +Cp = 78,740 ± 14120 pA·ms; *p* = 0.0078; *n* = 7; Fig. 4e, h) of spontaneous inhibitory postsynaptic currents (sIPSC) after 20 μM Cp application. Such reduction in the inhibitory input was not observed in slices without Cp treatment (frequency: control = 8.24 ± 1.20 Hz; −Cp = 9.24 ± 2.07 Hz; *p* = 0.5; *n* = 5; Fig. 4i, j; amplitude: control = 64.14 ± 10.50 pA; −Cp = 65.16 ± 10.68 pA; *p* = 0.5; *n* = 5; Fig. 4i, k; charge transfer: control = 106,100 ± 7577 pA·ms; −Cp = 105,100 ± 14700 pA·ms; *p* = 0.5; *n* = 5; Fig. 4i, l). These data support the Cp-induced desynchronization of PC activity observed in prior experiments (Fig. 2) since the inhibitory input to PCs plays an important role in the generation and maintenance of gamma oscillations [2, 3]. In addition, these results also suggest that Cp could induce a generalized reduction in neuronal activity including PCs and possibly also interneurons in hippocampal area CA3.

### Cp Induces an Increase in First-Spike Latency in PCs

**Fig. 5:**
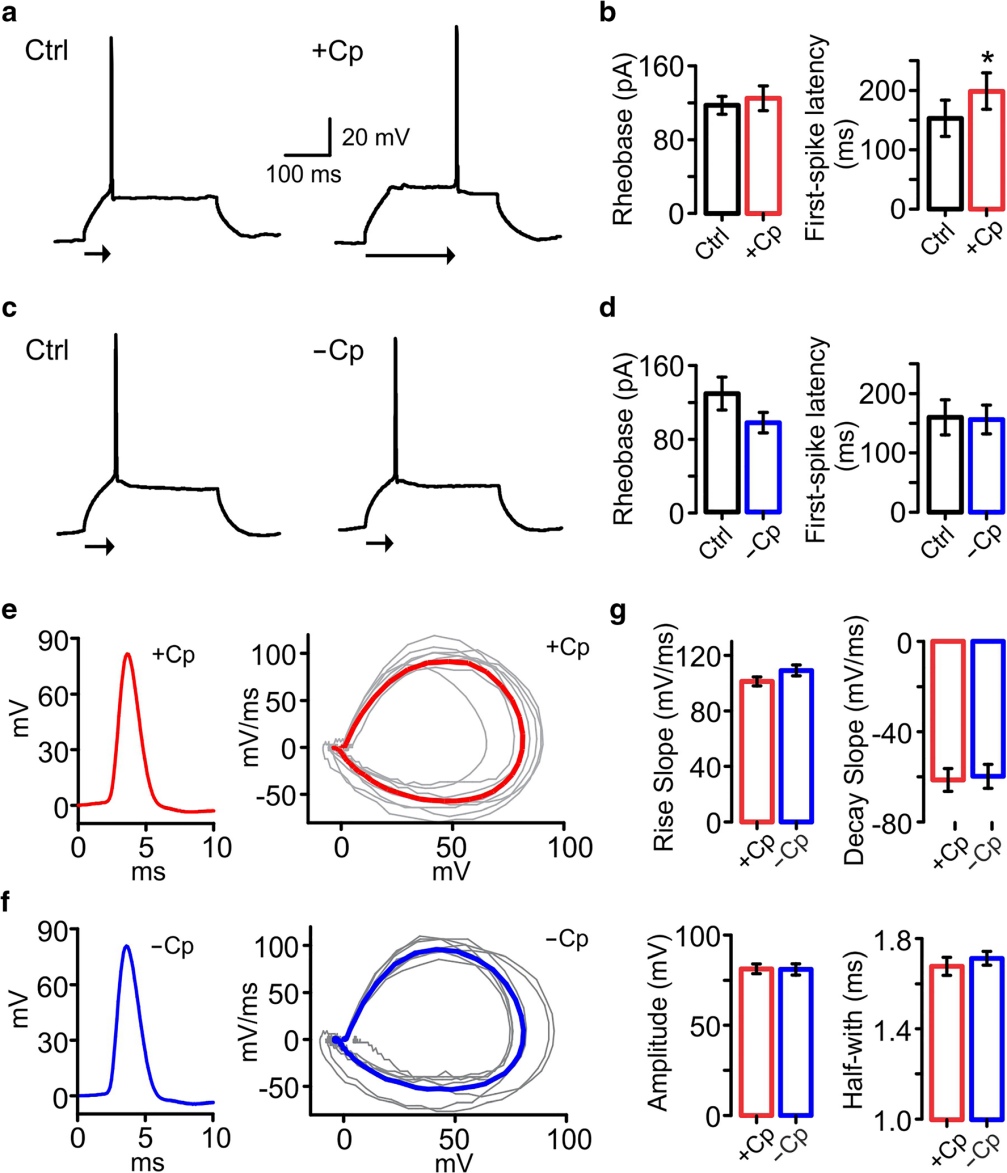
Capsaicin (Cp) induces an increase in the first-spike latency of CA3 PCs. **a** Representative traces of APs elicited in PCs with a minimum depolarizing current step (rheobase) before (Ctrl) and after 20 μM Cp treatment (+Cp, 20 min). Arrows below recordings indicate the time to the AP firing from the beginning of the current step (first-spike latency). **b** Summary bar graphs of the current threshold (rheobase) and first-spike latency before (Ctrl, black) and after 20 μM Cp treatment (+Cp, 20 min, red). **c** Representative traces of APs elicited in PCs with a minimum depolarizing current step (rheobase) in control slices without Cp treatment at the same time points as in **a** (Ctrl, −Cp 20 min). Arrows below recordings indicate the first-spike latency. **d** Summary bar graphs of the current threshold (rheobase) and first-spike latency in the experimental conditions describe in **c**. **e** Average AP from PCs in slices treated with 20 μM Cp (left, 20 min) and AP wave form graph (right) of each replicate (gray lines) and the average (red line). **f** Average AP from PCs in slices without Cp treatment at the same time point as **e** (20 min, left) and AP wave form graph (right) of each replicate (gray lines) and the average (blue line). **g** Summary bar graphs comparing AP waveform quantifications from slices with (red) and without (blue) Cp treatment at the same time point (20 min). Note that Cp induces a delay in the spiking time of PCs that is represented by the increase in the arrow length below the representative traces. Wilcoxon signed rank test (one-tailed; **b**, **d**) and Mann-Whitney test (one-tailed; **g)** were used for statistical significance on absolute values. Data is presented as mean ± SEM. *p < 0.05

Because the reduction in inhibitory input does not explain the Cp-induced reduction of AP firing rate observed in PC, we proceeded to investigate whether Cp induces an alteration in the AP firing threshold. We tested this possibility by implementing a rheobase protocol in current-clamp recordings consisting of injecting increasing current steps into PCs to elicit the firing of a single action potential (threshold-current). Our results showed that 20 μM Cp did not alter the threshold current since no changes were found in the rheobase (control = 116.9 ± 9.68 pA; +Cp = 124.4 ± 13.38 pA; *p* = 0.2002; *n* = 8; Fig. 5a, b). However, we found that Cp induced a delay in the first-spike latency (control = 153.1 ± 30.54 ms; +Cp = 198.8 ± 30.48 ms; *p* = 0.0195; *n* = 8; Fig. 5a, b). In contrast, no changes were observed in PCs from slices without Cp treatment: neither in rheobase (control = 127.9 ± 17.79 pA; −Cp = 96.43 ± 11.06 pA; *p* = 0.0538; *n* = 7; Fig. 5c, d) nor in first-spike latency (control = 160.0 ± 28.91 ms; −Cp = 156.5 ± 24.27 ms; *p* = 0.4688; Fig. 5c, d).

To test whether Cp could induce alterations in AP properties, we analyzed the waveform of PC APs from hippocampal slices with and without Cp treatment. As shown in Fig. 5, there were no differences in the AP waveform since no changes were found in the rise slope (+Cp = 101.3 ± 3.24 mV/ms: *n* = 8; −Cp = 109.3 ± 3.95 mV/ms: *n* = 8; *p* = 0.0657; Fig. 5e–g), decay slope (+Cp = −61.42 ± 5.07 mV/ms: *n* = 8; −Cp = −59.86 ± 5.33 mV/ms: *n* = 7; *p* = 0.4769; Fig. 5e–g), amplitude (+Cp = 81.4 ± 2.7 mV: *n* = 8; −Cp = 81.11 ± 3.12 mV: *n* = 7; *p* = 0.3472; Fig. 5e–g), and half-width (+Cp = 1.68 ± 0.04 ms: *n* = 8; −Cp = 1.72 ± 0.03 ms: *n* = 7; *p* = 0.2317; Fig. 5e–g). All together, these data indicate that the Cp-induced reduction of AP firing rate is related to the increase in the first-spike latency.

### Cp-Induced Increase in First-Spike Latency Involves Activation of Na^+^/K^+^-ATPase

**Fig. 6:**
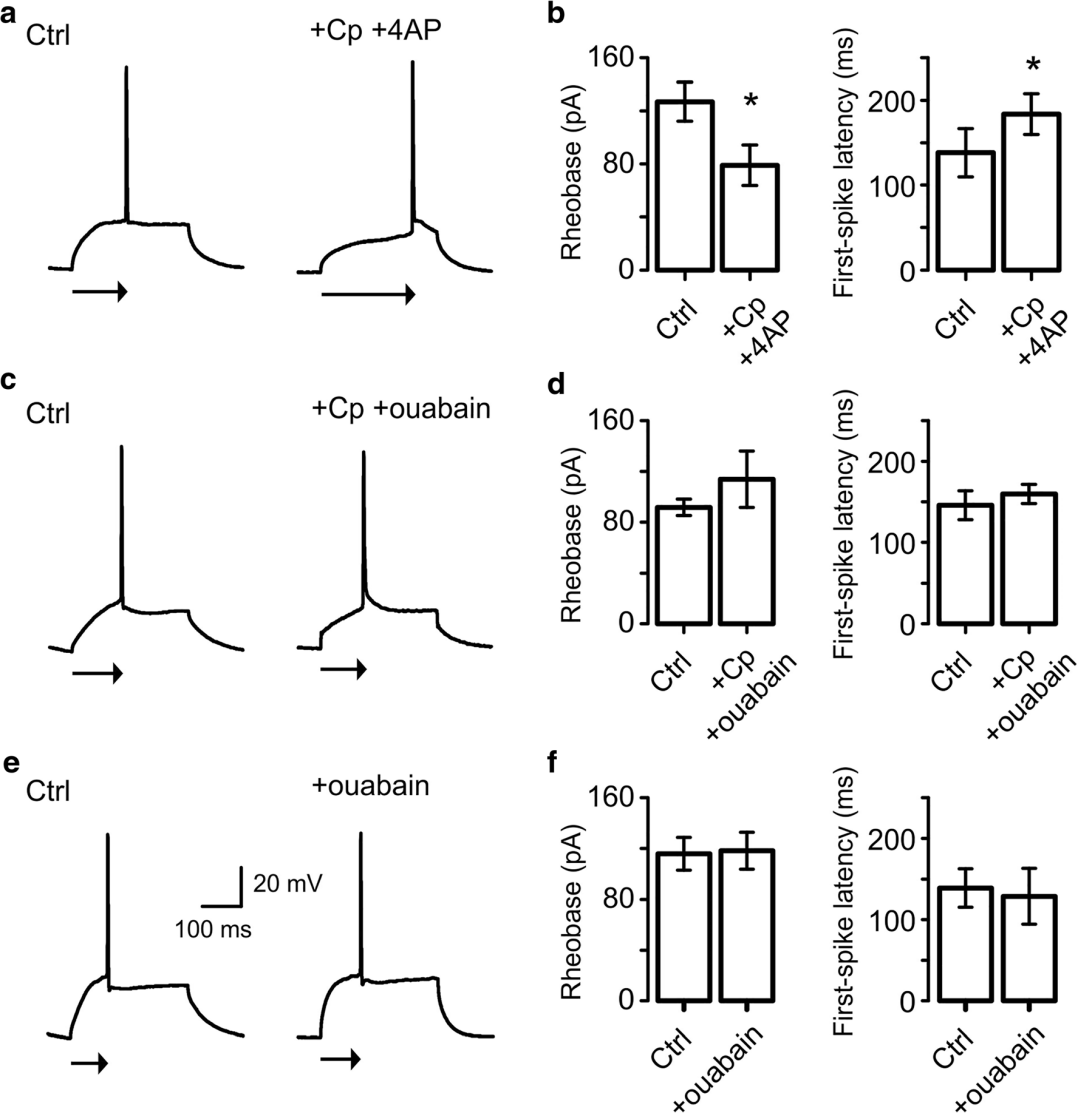
Capsaicin (Cp)-induced increase in first-spike latency involves activation of Na^+^/K^+^-ATPase. **a** Representative traces of APs elicited in PCs with a minimum depolarizing current step (rheobase) before (Ctrl) and after 20 μM Cp plus 30 μM 4AP treatment (+Cp +4-AP, 20 min). Arrows below recordings indicate the time to the AP firing from the beginning of the current step (first-spike latency). **b** Summary bar graphs of the current threshold (rheobase) and first-spike latency quantifications of the experimental conditions described in **a**. **c** Representative traces of APs elicited in PCs with a rheobase current before (Ctrl) and after 20 μM Cp plus 25 μM ouabain treatment (+Cp +ouabain, 20 min). Arrows below recordings indicate the time to the AP firing from the beginning of the current step. **d** Summary bar graph of the current threshold (rheobase) and first-spike latency quantifications of the experimental conditions describe in **c**. **e** Representative traces of APs elicited in PCs with a rheobase current before (Ctrl) and after 25 μM ouabain application (+ouabain, 20 min). Arrows below recordings indicate the first-spike latency. **f** Summary bar graph of the current threshold (rheobase) and first-spike latency quantifications of the experimental conditions describe in **e**. Note that ouabain, but not 4-AP, blocks the Cp-induced increase in the spiking time of PCs (arrows) suggesting a role of Na^+^/K^+^-ATPase in the Cp effects on the hippocampal neuronal network. Wilcoxon signed rank test (one-tailed) was used for statistical significance on absolute values. Data is presented as mean ± SEM. *p < 0.05

It has been reported that first-spike latency can be regulated by the voltage-dependent potassium current D (*I*_D_) [31–33]. To explore this possibility, we performed experiments using a low 4-AP concentration (30 μM) known to selectively inhibit *I*_D_ [31, 32]. We synaptically isolated PCs by bath application of 50 μM AP5, 20 μM DNQX, and 50 μM picrotoxin at least 15 min before recording in order to prevent a change in the first-spike latency due to the increase in spontaneous synaptic activity by 4-AP application. Interestingly, we found that despite the fact that 30 μM 4-AP application, together with 20 μM Cp, induced a decrease in the threshold current (rheobase: control = 127.0 ± 14.71 pA; +Cp +4-AP = 79.0 ± 15.28 pA; *p* = 0.0267; *n* = 5; Fig. 6a, b), 4-AP did not prevent the increase in the first-spike latency induced by Cp (control = 138.3 ± 28.55 ms; +Cp +4-AP = 183.9 ± 23.99 ms; *p* = 0.0313; *n* = 5; Fig. 6a, b). As our previous data suggested, these results indicate that modulation of *I*_D_ does not underlie the AP firing reduction induced by Cp.

Another mechanism that could be regulating the first-spike latency involves the Na^+^/K^+^-ATPase [33]. To test this possibility, we performed recordings using the Na^+^/K^+^-ATPase blocker ouabain together with Cp on synaptically isolated PCs. We decided to use a 25 μM ouabain concentration to block all Na^+^/K^+^-ATPase isoforms [34]. As shown in Fig. 6, the combined treatment (25 μM ouabain + 20 μM Cp) did not induce alterations in the threshold current (rheobase; control = 91.67 ± 6.54 pA; +Cp +ouabain = 113.9 ± 22.2 pA; *p* = 0.2188; *n* = 6; Fig. 6c, d) but completely prevented the increase in the first-spike latency (control = 145.9 ± 17.9 ms; +Cp +ouabain = 159.7 ± 11.78 ms; *p* = 0.2188; *n* = 6; Fig. 6c, d). In control experiments, ouabain application alone (25 μM) did not alter either the threshold current (rheobase: control = 116.3 ± 12.97 pA; +ouabain = 118.8 ± 14.63 pA; *p* = 0.4375; *n* = 4; Fig. 6e, f) or the first-spike latency (control = 139.5 ± 23.62 ms; +ouabain = 129.2 ± 34.37 ms; *p* = 0.3125; *n* = 4; Fig. 6e, f).

Taken together, our data demonstrate that Cp, independently of TrpV1 activation, induces a reduction in AP firing rate and desynchronization of PC activity in the hippocampal network involving the Na^+^/K^+^-ATPase and that this mechanism is responsible for the Cp-induced impairment of functional network dynamics in mouse hippocampus.

Finally, we studied the effect of ouabain on ongoing gamma oscillations in order to test whether Na^+^/K^+^-ATPase inhibition could prevent the Cp-induced reduction of gamma oscillation power. We found that 25 μM ouabain application induced a fast time-dependent reduction of gamma oscillations (Fig. S3D, Table 4). Gamma oscillations were completely inhibited after 7 min of ouabain application (Fig. S3A–E) demonstrating that it is not feasible to directly test the role of Na^+^/K^+^-ATPase by electrophysiological means.

**Table 4:** Summary of the time course of gamma oscillation power after ouabain application

Time (min)	Integrated gamma power (10^−9^ V^2^)
	+ouabain (*n* = 7)
Average (− 5 to − 1)	2.26 ± 0.49
0	2.31 ± 0.59 (*p* = 0.3125)
1	2.22 ± 0.59 (*p* = 0.3125)
2	*1.62 ± 0.29 (*p* = 0.0313)
3	*1.10 ± 0.24 (*p* = 0.0313)
4	*0.51 ± 0.12 (*p* = 0.0313)
5	*0.27 ± 0.08 (*p* = 0.0313)
6	*0.12 ± 0.03 (*p* = 0.0313)
7	*0.05 ± 0.01 (*p* = 0.0313)
8	*0.03 ± 0.007 (*p* = 0.0313)
9	*0.04 ± 0.007 (*p* = 0.0313)

For statistical analysis, the value of each time point was compared to the average of its own 5 min of control activity. Wilcoxon signed rank test (one-tailed) was used for statistical significance on absolute values. Data is presented as mean ± SEM

*p < 0.05

## Discussion

In the peripheral nervous system, it is well documented that Cp’s TrpV1-independent effects are concentration-dependent [13–15]. For instance, in trigeminal ganglion sensory neurons, it has been reported that a lower Cp concentration (1 μM) can induce a slight inhibition of the transient potassium current (*I*_A_) and sustained potassium current (*I*_K_) [13, 14]. Such Cp TrpV1-independent inhibition of *I*_A_ and *I*_K_ increases with higher Cp concentrations (10 to 100 μM) [14]. Even though in the present study we use a higher Cp concentration to explore its TrpV1-independent effects compared to our previous study [24], our previous observations already raised the possibility that Cp had TrpV1-independent effects at concentrations normally reported to specifically activate TrpV1in CNS (10 μM) [12, 16, 17]. Supporting our data, using TrpV1 KO mice, Benninger and coworkers (2008) [23] also showed that 10 μM Cp induced a reduction in the amplitude of evoked EPSC in dentate gyrus. Interestingly, they also found that Cp induced an increased in frequency, but not amplitude, of spontaneous EPSCs in granular cells [23], which is in contrast with the lack of effect of Cp on spontaneous EPSCs we observed in CA3 PCs. This suggests a difference in the sensitivity of various cell types to the TprV1-independent effect of Cp.

Previously, we have described that 10 μM Cp can activate a TrpV1-dependent as well as a TrpV1-independent pathway [24]. Here, we found that a slightly higher concentration (20 μM) activates specifically the TrpV1-independent pathway since the application of TrpV1 antagonist Cz did not block the effects caused by 20 μM Cp. This specific activation of the TrpV1-independent pathway can be explained taking in consideration three main points: (1) the low expression of TrpV1 receptor in hippocampus compared to other brain structures such as cortex [35–39] and the fact that TrpV1 expression in hippocampal PCs seems to be present in the cytoplasm rather than the cell membrane [40]. Thus, the TrpV1 receptor activation in our experimental conditions would be very small and is completely occluded by the TrpV1-independent pathway activated by Cp. (2) The desensitization of the TrpV1 receptor [41] suggests that the small receptor pool expressed on the cell membrane is quickly inactivated and thus capsaicin essentially activates only the TrpV1-independent pathway. (3) A pathology-selective activation of the TrpV1-dependent vs. the TrpV1-independent pathway in which TrpV1 expression may be upregulated under pathological conditions only. This hypothesis is supported by our previous study in which we found that capsaicin activates the TrpV1 receptor only under the pathological conditions triggered by the Alzheimer’s disease-related peptide amyloid-β while in physiological conditions, the TrpV1-independent mechanism is activated [24]. Further support for this hypothesis comes from other studies showing that TrpV1 expression in hippocampus is increased in animal models of epilepsy [42–44].

The results shown here suggest that the mechanism underlying the TrpV1-independent effects of Cp on cognition-relevant functional network dynamics such as gamma oscillations is related to a reduction in the AP firing rate driven by an effect on spike-timing in PCs, seen as an increase in the first-spike latency. First-spike latency in response to a stimulus in sensory neurons has been shown to play an important role in the propagation/transmission of information in different sensory systems [45–49]. For instance, a stimulus parameter like the location of a sound can modulate the delay of the first-spike latency [48]. In hippocampus, modulation of spike-timing plays an important role in neuronal network synchronization of AP firing facilitating the generation of synchronized activity such as gamma oscillations [2, 3]. Our data is in accordance with this assumption since we show that spike-timing alteration by Cp can desynchronize PCs AP firing resulting in a reduction of gamma oscillation power and rhythmicity.

Several studies have reported that the activation of the slowly inactivating current *I*_D_ is responsible for the delay in the first-spike latency in hippocampal neurons [31, 32, 50]. We found that despite Cp-induced increase in the first-spike latency on PCs, this effect was not related to an increase in *I*_D_ conductance since blocking *I*_D_ with 4-AP did not reverse the Cp effect. Instead, our data show that Cp-induced increase in the first-spike latency could be reversed by ouabain, demonstrating the involvement of the Na^+^/K^+^-ATPase in the Cp-induced reduction of AP firing rate and the subsequent reduction in gamma oscillations. Supporting our conclusion, Na^+^/K^+^-ATPase has been reported to regulate excitability in several neuron types, as well as rhythmic network activity in the CNS, working as a sensor of AP firing activity in neurons in which Na^+^/K^+^-ATPase function increases after prolonged or intense AP firing due to intracellular Na^+^ accumulation [33, 34, 51–54]. The reduction of excitability by activity-dependent recruitment of the Na^+^/K^+^-ATPase has been studied in hippocampus by Gulledge and coworkers (2013) [34]. They reported that after trains of APs in CA1 PCs, a long-lasting sodium-dependent afterhyperpolarization (AHP) mediated by Na^+^/K^+^-ATPase was observed. By applying a minimally suprathreshold current to elicit the firing of a single AP (rheobase protocol), they showed that the Na^+^/K^+^-ATPase-dependent AHP induction inhibited the generation of APs for an average of 7 s. Moreover, similar to our data, when they increased the intensity of the suprathreshold current to force the neuron to fire after the Na^+^/K^+^-ATPase-dependent AHP induction, an increase in the first-spike latency was observed [33]. We suggest that a similar mechanism could underlie the Cp-induced reduction of AP firing rate observed in our study.

In addition, we found that gamma oscillations were completely inhibited after 7 min of ouabain treatment. It is well known that Na^+^/K^+^-ATPase plays an important role in setting the resting membrane potential [55]. Because the ouabain concentration used in this study (25 μM) inhibits all Na^+^/K^+^-ATPase isoforms, an imbalance in the ionic gradient responsable for the maintance of the membrane potential is created. Thus, neurons in the hippocampal network fail to repolarize and eventually stop firing. The inhibition of PC firing may underlie the ouabain-related inhibition of gamma oscillations observed in this study.

Although our data strongly suggest that the TrpV1-independent effects of Cp on AP firing and the subsequent reduction in gamma oscillations involves the activation of the Na^+^/K^+^-ATPase, the mechanism underlying such activation remains unclear. It is possible that Cp regulates the activity of Na^+^/K^+^-ATPase by a direct interaction or, perhaps more likely, that Cp acts through the activation of an intracellular regulatory mechanisms upstream of Na^+^/K^+^-ATPase. The activity of Na^+^/K^+^-ATPase is regulated by phosphorylation by PKA and PKC kinases, especially in the subunit FXYD1, which is highly expressed in the brain. FXYD1 is an inhibitory subunit and its phosphorylation releases the inhibition and increases Na^+^/K^+^-ATPase activity [56, 57]. It is possible then that Cp can regulate Na^+^/K^+^-ATPase activity through recruiting these kinases and increasing the phosphorylation of the FXYD1 subunit. To test such a hypothesis is beyond the focus of this study but opens the door for future experiments in order to ascertain whether the phosphorylation levels of subunit FXYD1 change following Cp treatment and to study the exact molecular pathway responsible for such an effect.

We also found that Cp induced a reduction in inhibitory input to PCs by reducing the frequency and amplitude of sIPSCs, indicating that Cp has pre- and post-synaptic effects. Moreover, our data also suggest that Na^+^/K^+^-ATPase recruitment by Cp could also impair CA3 interneuron activity, which is important for the generation of gamma oscillations [2, 3]. This possibility is supported by a study from Richards and coworkers (2007) [35] showing that Na^+^/K^+^-ATPase inhibition can regulate inhibitory input to PCs in the subiculum. They found that interneuron-Na^+^/K^+^-ATPase inhibition by a low nanomolar ouabain concentration elicited an increase in frequency but not amplitude of sIPSCs recorded in PCs [34]. Taken together, these studies suggest that Cp recruitment of Na^+^/K^+^-ATPase can reduce the excitability of PCs, and possibly also interneurons, inducing a reduction of synchrony in the hippocampal neuronal network and thus decreasing cognition-relevant network activity such as gamma oscillations.

Dysfunction of gamma oscillations in the brain is a hallmark of several psychiatric and neurologic disorders such as Alzheimer disease, attention deficit hyperactivity disorder (ADHD), schizophrenia, and epilepsy [58]. In some of them, patients exhibit decreased gamma oscillation power (AD, ADHD) while in others, gamma power may be higher compared to healthy subjects (schizophrenia, epilepsy) [58, 59]. Based on the results of the present study as well as data from other labs, it would be interesting to investigate Cp as a putative treatment aimed at restoring the enhanced power of gamma oscillations back to normal values by using mouse models of schizophrenia and epilepsy both in vitro and in vivo. The feasibility of this hypothesis is supported by (1) the correlation between the increase in gamma oscillations and the positive symptoms like hallucinations in schizophrenia patients [60] and the acute psychosis behavior in a pharmacological murine model of schizophrenia [61], and (2) typical and atypical antipsychotic drug administration reduced the increase in gamma oscillations displayed in the pharmacological murine model of schizophrenia [61]. Thus, the use of Cp to restore the power of gamma oscillations to healthy control levels is a suitable strategy in the fight against these brain disorders.

In conclusion, our data strongly suggest that the mechanism underlying the TrpV1-independent effects of Cp on functional network dynamics is related to a reduction in AP firing rate. Our results also show that the reduction in AP firing rate is coupled to an increase in the first-spike latency and involves a Cp-dependent recruitment of the Na^+^/K^+^-ATPase. Since growing evidence suggests that TrpV1 is involved in several brain functions by modulating glial and neuronal activity in normal and pathological conditions, our data is also important to consider in the context of the use of Cp as a tool to study TrpV1 function in the CNS.

## Electronic supplementary material

Fig. S1 **Capsazepine (Cz) and DMSO effects on gamma oscillations. A-B)** Representative sample traces (top) power spectra (middle) and auto-correlograms (bottom) of KA-induced gamma oscillations in hippocampal slices in control conditions and after 20 min treatment with 20 μM capsazepine (+Cz, purple) or 0.05% DMSO (+DMSO, blue). **C)** Time-course of the integrated power of gamma oscillations from slices treated with Cz and slices treated with DMSO. **D)** Summary bar-graphs of the integrated gamma power and coefficient of rhythmicity (Cr), respectively, from the experimental conditions described in A-B. DMSO final concentration in the bath (0.05%) corresponds to the highest concentration used with the combined Cp +Cz treatment. Integrated power was measured on 1 min segments every 5 min after treatment application. Power quantification was performed after 20 min of treatment application and compared to the average of 5 min of control activity. Cr quantification was performed between the 20 min time point and 1 min of control activity before treatment application. Wilcoxon signed rank test (one-tailed) was used for statistical significance on absolute values. Data is presented as mean ± SEM. * indicates *p* < 0.05. (PNG 723 kb)

High Resolution Image (TIF 1.80 mb)

Fig. S2 **Capsaicin (Cp)-induced reduction in AP-firing rate at 34 °C. A)** Representative recordings of the AP-firing rate of PCs from slices not activated with KA and recorded at 34 °C **B)** Time-course of AP-firing rate recorded at 34 °C. **C)** Summary bar-graph of the experimental condition described in A. Note that 20 μM Cp (+Cp, 20 min) induced a reduction in the PC firing rate recorded at 34 °C similar to PCs in slices at room temperature (Fig. 3). AP-firing rate quantification was performed on 1 min segments after 20 min of treatment application and compared to the average of 5 min of control activity. Wilcoxon signed rank test (one-tailed) was used for statistical significance on absolute values. Data is presented as mean ± SEM. * indicates p < 0.05. (PNG 191 kb)

High Resolution Image (TIF 555 kb)

Fig. S3 **Ouabain effect on gamma oscillations. A)** Representative sample traces of KA-induced gamma oscillations in hippocampal slices in control conditions and after 9 min treatment with 25 μM ouabain (+ouabain). **B)** Power spectra and **C)** auto-correlograms of the KA-induced gamma oscillations from the experimental condition described in A. **D)** Time-course of the normalized integrated power of gamma oscillations from the experimental conditions described in A showing the time-dependent decrease in gamma power in slices treated with ouabain (gray line). **E)** Summary bar-graph of the integrated gamma power in control conditions and after 9 min treatment with 25 μM ouabain (+ouabain). Integrated power was measured on 1 min segments. Power quantification was performed after 9 min of treatment application and compared to the average of 5 min of control activity. Wilcoxon signed rank test (one-tailed) was used for statistical significance on absolute values. Data is presented as mean ± SEM. * indicates p < 0.05 and ** indicates *p* < 0.01. (PNG 414 kb)

High Resolution Image (TIF 943 kb)
