# Supplementary figures and images for: Capsaicin-Induced Impairment of Functional Network Dynamics in Mouse Hippocampus via a TrpV1 Receptor-Independent Pathway: Putative Involvement of Na+/K+-ATPase
Source: Mol Neurobiol. 2019 Nov 7;57(2):1170–85. doi: 10.1007/s12035-019-01779-3 (PMC7031213; doi:10.1007/s12035-019-01779-3)

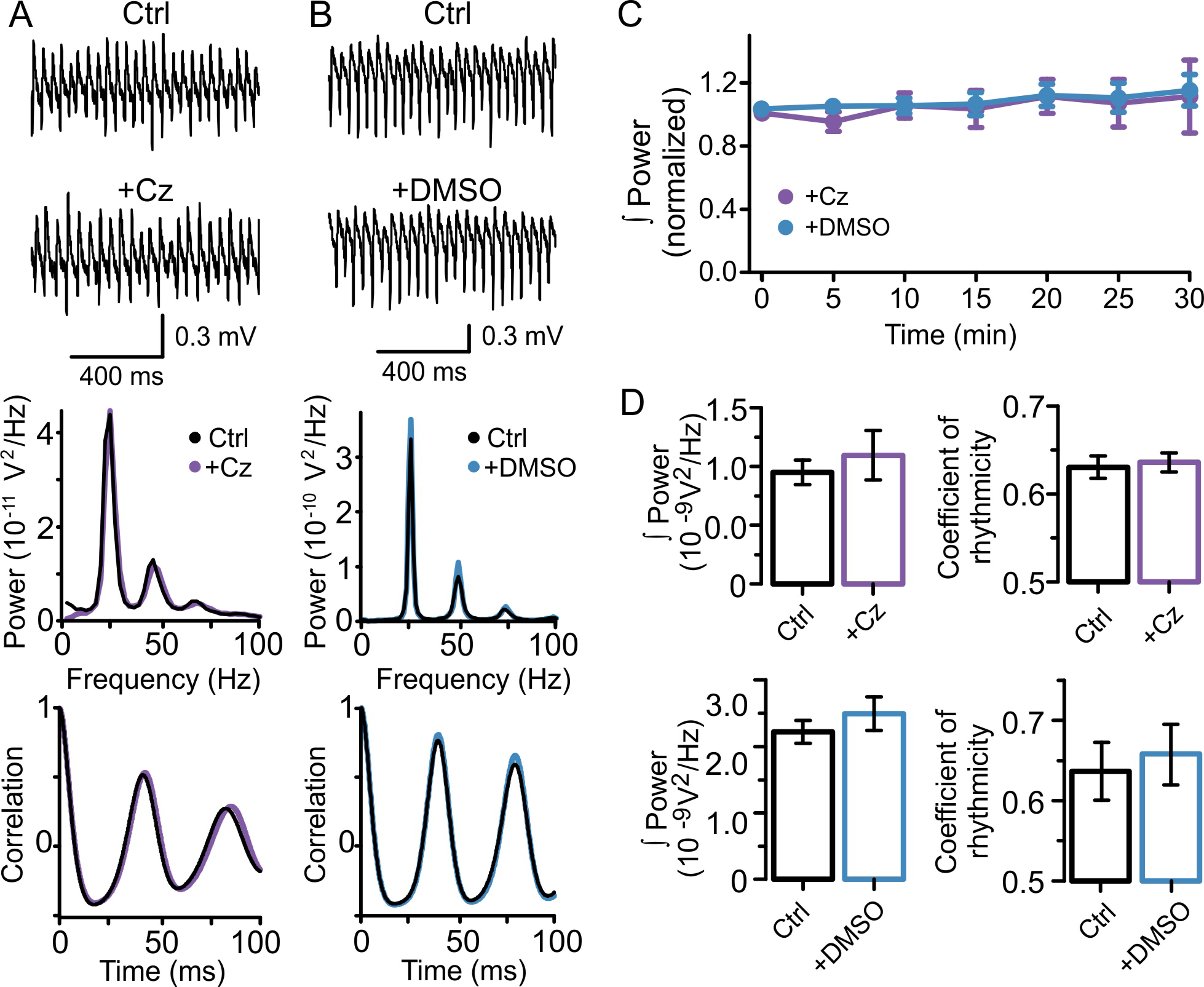

Supplement: Supplementary file 1 — Capsazepine (Cz) and DMSO effects on gamma oscillations. A-B) Representative sample traces (top) power spectra (middle) and auto-correlograms (bottom) of KA-induced gamma oscillations in hippocampal slices in control conditions and after 20 min treatment with 20 μM capsazepine (+Cz, purple) or 0.05% DMSO (+DMSO, blue). C) Time-course of the integrated power of gamma oscillations from slices treated with Cz and slices treated with DMSO. D) Summary bar-graphs of the integrated gamma power and coefficient of rhythmicity (Cr), respectively, from the experimental conditions described in A-B. DMSO final concentration in the bath (0.05%) corresponds to the highest concentration used with the combined Cp +Cz treatment. Integrated power was measured on 1 min segments every 5 min after treatment application. Power quantification was performed after 20 min of treatment application and compared to the average of 5 min of control activity. Cr quantification was performed between the 20 min time point and 1 min of control activity before treatment application. Wilcoxon signed rank test (one-tailed) was used for statistical significance on absolute values. Data is presented as mean ± SEM. * indicates p < 0.05. (PNG 723 kb) [file 12035_2019_1779_Fig7_ESM.png]

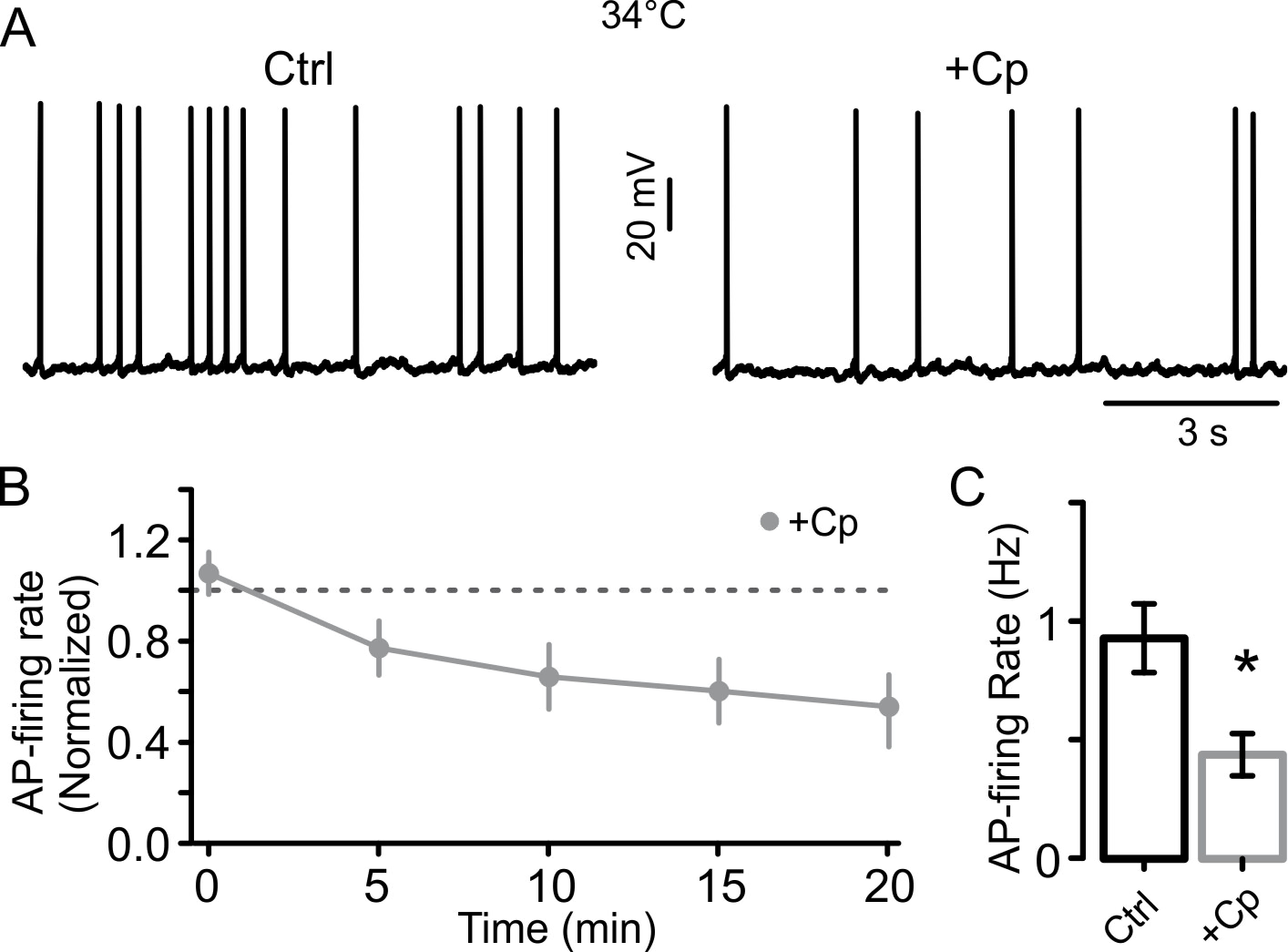

Supplement: Supplementary file 3 — Capsaicin (Cp)-induced reduction in AP-firing rate at 34 °C. A) Representative recordings of the AP-firing rate of PCs from slices not activated with KA and recorded at 34 °C B) Time-course of AP-firing rate recorded at 34 °C. C) Summary bar-graph of the experimental condition described in A. Note that 20 μM Cp (+Cp, 20 min) induced a reduction in the PC firing rate recorded at 34 °C similar to PCs in slices at room temperature (Fig. 3). AP-firing rate quantification was performed on 1 min segments after 20 min of treatment application and compared to the average of 5 min of control activity. Wilcoxon signed rank test (one-tailed) was used for statistical significance on absolute values. Data is presented as mean ± SEM. * indicates p < 0.05. (PNG 191 kb) [file 12035_2019_1779_Fig8_ESM.png]

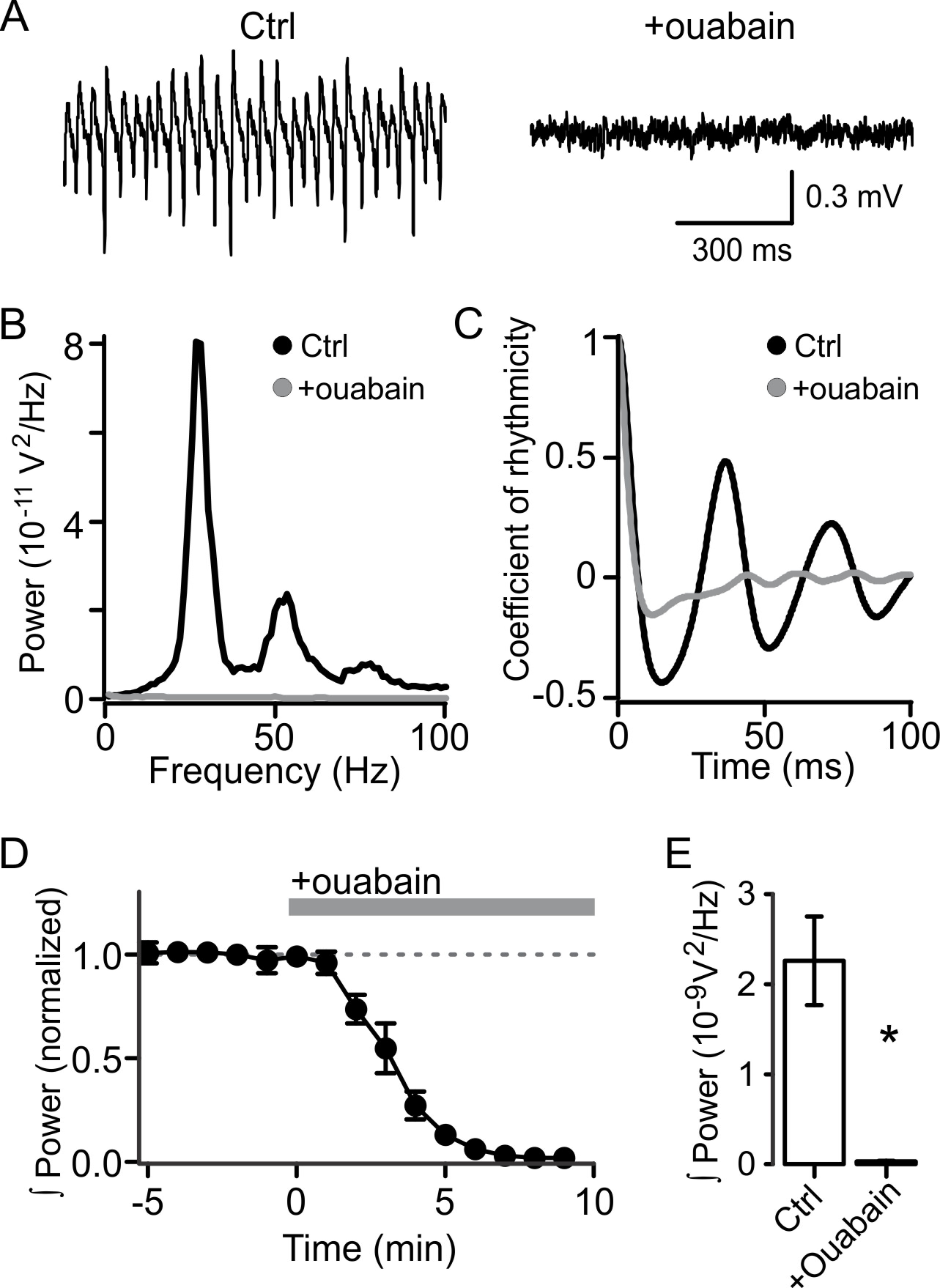

Supplement: Supplementary file 5 — Ouabain effect on gamma oscillations. A) Representative sample traces of KA-induced gamma oscillations in hippocampal slices in control conditions and after 9 min treatment with 25 μM ouabain (+ouabain). B) Power spectra and C) auto-correlograms of the KA-induced gamma oscillations from the experimental condition described in A. D) Time-course of the normalized integrated power of gamma oscillations from the experimental conditions described in A showing the time-dependent decrease in gamma power in slices treated with ouabain (gray line). E) Summary bar-graph of the integrated gamma power in control conditions and after 9 min treatment with 25 μM ouabain (+ouabain). Integrated power was measured on 1 min segments. Power quantification was performed after 9 min of treatment application and compared to the average of 5 min of control activity. Wilcoxon signed rank test (one-tailed) was used for statistical significance on absolute values. Data is presented as mean ± SEM. * indicates p < 0.05 and ** indicates p < 0.01. (PNG 414 kb) [file 12035_2019_1779_Fig9_ESM.png]
